# *Salvia miltiorrhiza* hydroalcoholic extract inhibits postoperative peritoneal adhesions in rats

**DOI:** 10.1186/s12906-021-03300-7

**Published:** 2021-04-20

**Authors:** Abbas Raisi, Omid Dezfoulian, Farshid Davoodi, Shayan Taheri, Soroush Afshar Ghahremani

**Affiliations:** 1 Department of Clinical Sciences, Faculty of Veterinary Medicine, Lorestan University, Khorramabad, Iran; 2 Department of Pathobiology, Faculty of Veterinary Medicine, Lorestan University, Khorramabad, Iran

**Keywords:** Salvia miltiorrhiza, Peritoneal adhesions, Postoperative adhesions, Antioxidants

## Abstract

**Background:**

One of the most prevalent postoperative complications is believed to be intra-abdominal peritoneal adhesions, which is followed by several complications. Several adhesion prevention products have been examined, yet none of them were found to be completely effective. The current research is conducted to evaluate the beneficial effects of *Salvia miltiorrhiza* hydroalcoholic extract in inhibiting postoperative peritoneal adhesions in rats.

**Methods:**

Forty rats were randomly classified into five equal groups (*n* = 8): 1) the normal group did not undergo surgical operations, 2) the control group in which the adhesion was induced, and which did not receive any treatment, 3) distilled water group that received distilled water, and 4,5) treatment groups treated with 1 and 5% of *Salvia miltiorrhiza* hydroalcoholic extract. The rats were euthanized 14 days following the surgery and the macroscopic score, the microscopic score of granulomatous inflammation and granulation tissue formation, IHC markers (vimentin, CD31, IL-1β, COX-2, and iNOS), and oxidative stress biomarkers (MDA, GPx, CAT, and TAC) were assessed in the experimental groups of the study.

**Results:**

The difference between the control group and other groups for the adhesions macroscopic score, microscopic score, IHC markers, and oxidative stress biomarkers was significant (*p* < 0.05). Distilled water had no protective effect on the formation of peritoneal adhesions. *Salvia miltiorrhiza* treatment in two different doses significantly reduced macroscopic and microscopic scores, MDA concentration, Vimentin, IL-1β, COX-2, and iNOS compared to the control group (*p* < 0.05). The levels of GPx, CAT, and TAC in the treatment groups increased significantly compared with the control group (*p* < 0.05). Our findings revealed that a higher dose of *Salvia miltiorrhiza* was more effective in reducing peritoneal adhesions, proinflammatory and mesenchymal cell markers, and oxidative stress.

**Conclusions:**

*Salvia miltiorrhiza* extract, owing to its strong antioxidant and anti-inflammatory properties, could effectively reduce peritoneal adhesions. Therefore, *Salvia miltiorrhiza* is recommended to be used as an effective anti-peritoneal post-operative adhesive agent.

## Background

Peritoneal adhesion are categorized as the major abdominal surgical complications usually triggered by peritoneum injuries [1, 2]. Adhesions attach the intra-abdominal organs to the peritoneum, and following a laparotomy, they develop in 95% of patients [3]. The adhesion process is essential for the healing of the peritoneum, but sometimes becomes problematic and causes obstruction in the small intestine, intra-abdominal pain, fistula formation, and female subfertility and infertility [4, 5]. Since approximately 50% of hospital referrals after surgical operations are due to adhesion-related complications, there has always been a great deal of effort to prevent them and numerous studies have been carried out in this regard [6, 7].

Trauma, ischemia, infection, thermal injury and foreign bodies are the most prevalent factors involved in the formation of the postoperative peritoneal adhesions [8]. Adhesion development is the consequence of inflammatory reactions, fibrin formation, and activation of matrix metalloproteinases (MMPs) [9]. Once the peritoneum is damaged, inflammatory mediators and cytokines, such as interleukin-1β (IL-1β), interleukin-6 (IL-6), tumor necrosis factor-alpha (TNF-α), and some growth factors are released, which stimulate mesothelial cell growth, angiogenesis, and fibroblast aggregation and cause adhesion bands [10]. The employed methods to prevent adhesions mentioned in previous studies include the use of laparoscopy instead of laparotomy, the use of antibiotics, surgical adjuvants, Thrombolytic drugs, NSAIDs, corticosteroids, and Intraperitoneal Instillations [11].

*Salvia miltiorrhiza* is a famous medicinal plant with plenty of recognized properties, including antioxidants, anti-inflammatory, antimicrobials, anti-spasmodic, reduction of myocardial infarction, and platelet aggregation [12]. The genus Salvia possesses approximately 900 species, 58 of which are found in Iran, and certain species are native [12]. Beneficial effects of this plant have been proven in previous studies in cardiovascular disease [13, 14], renal dysfunction [15], liver fibrosis [16], Alzheimer’s disease [17], Parkinson’s disease [18], ischemic disorders in various organs including heart [19], and liver [16]. In a previous study, the *Salvia officinalis* essential oil was reported to be able to improve the healing of infectious wounds [20]. The anti-inflammatory effects of *Salvia miltiorrhiza* are owing to the inhibition of IL-1β, IL-6, IL-8, and TNF-α [21, 22]. Furthermore, it has been proven that rosmarinic acid and isosalvianolic acid are responsible for the inhibition of TNF-α, IL-1β, and IL-6 expression [21].

Effects of the *Salvia miltiorrhiza* stems and leaves total phenolic acids extract and roots and rhizome tanshinone extract in a mice model of the dextran sulfate sodium-induced colitis was investigated and it was concluded that *Salvia miltiorhiza* effectively inhibited TLR4/PI3K/AKT/mTOR signaling-related proteins that indicate the potent anti-inflammatory effects of the *Salvia miltiorrhiza* [23]. Cryptotanshinone, a natural product isolated from the Salvia miltiorrhiza, has been proven to significantly inhibit expression of RIP3, iNOS, NF-κB p65, COX-2, TNF-α, IL 6 in a mice model of ulcerative colitis suggesting the anti-inflammatory effects of the Cryptotanshinone [24]. Protocatechuic aldehyde from *Salvia miltiorrhiza* extract inhibited TNF-α and IL-6. It also reduced phosphorylation of MAPKs kinase and NF-κB in C57 mice [25]. Furthermore, Tanshinones and diethyl blechnics derived of *Salvia miltiorrhiza* extract were evaluated for anti-inflammatory effects and it was found that extract usage significantly reduced NO, TNF- α, IL 6, iNOS, COX-2, and NF-κB in the macrophage cells in vitro [26]. *Salvia miltiorrhiza* has been found to promote fibrinolysis and prevents platelet aggregation [27]. *Salvia miltiorrhiza* methanolic extract revealed high fibrinolytic activity in comparison to plasmin in vitro [28]. *Salvia miltiorrhiza* has been reported to reduce MDA levels following renal ischemia/reperfusion in rats [15]. Moreover, oxidative damage following testicular ischemia/reperfusion was investigated in rats and the hydroalcoholic extract of *Salvia miltiorrhiza* diminished MDA levels and increased GPx, CAT, and TAC levels indicating the effective antioxidant properties for this plant [29]. The antimicrobial effects of the hydroalcoholic extract of *Salvia miltiorrhiza* were evaluated in an investigation whose results indicated that the tanshinones and phenolic acids in the extract could inhibit the growth of many bacteria, particularly plant pathogens [30]. In another study, the anti-cytotoxic and antimicrobial effects of *Salvia miltiorrhiza* extract against Gram-negative, Gram-positive, and fungi were identified [31].

Previous studies demonstrated inflammatory cell reactions and deposition of peritoneal fibrin as the main pathophysiology of the adhesion formation [32, 33]. Hence, based on the potent anti-inflammatory and fibrinolytic effects of the *Salvia miltiorrhiza*, this research was designed to investigate the protective effects of *Salvia miltiorrhiza* hydroalcoholic extract in preventing post-surgery intraperitoneal adhesions.

## Materials and methods

### Extract preparation

To prepare the extract of *Salvia miltiorrhiza*, this plant was provided from Barij Essence research farm, Kashan, Iran. Relevant institutional permissions to collect *Salvia miltiorrhiza* were obtained. The use of plant parts in the present study complies with the international, national and/or institutional guidelines. The specimens were identified by botanical specialists of the biological department of Barij Essence research center, and deposited at the herbarium of the of Barij Essence research center, department of agriculture, Kashan, Iran (Herbarium No. 186–1). The plants were kept in a room at 25 °C until dried up completely. Afterwards, 400 g of the dried leaves were removed and added to 1 L of 70% hydro ethanol and maintained at laboratory temperature for 2 days. Thereafter, the liquid extract analysis was carried out (Code: FCL64–03, the central laboratory of Barij Essence Pharmaceutical Co, Kashan, Iran). The results of the liquid analysis are represented in Table 1. A paper filter was utilized to filter the extract and it was concentrated using a rotary evaporator (IKA RV 3 V Rotary Evaporator, Staufen, Germany). The resulted powder was then stored at − 20 °C until being used in the experiments. 1 and 5% solutions were prepared by dissolving the extract powder in distilled water and employed in subsequent experiments.

**Table 1:** Salvia miltiorrhiza hydroalcoholic extract analyze (code NO. FCL64–03, Central Laboratory of the BARIJ ESSENCE PHARMACEUTICAL CO. Kashan, Iran)

Test	Result	Reference method
Specific Gravity (g/ml)	0.974	USP38-‹841›
pH	5.83	ISIRI1487-(2–2-5)
Dry residue (%w/w)	12.83	BP2015
Refractive Index (nD)	1.3764	ISIRI2274–6
Rosmarinic acid (mg/ml)	14.28	Barij Essence reference method
Color	Brown	Barij Essence reference method
Clarity	Clear	Barij Essence reference method
Odor	Special	Barij Essence reference method

### Ferric reducing-antioxidant power (FRAP) analysis

The total antioxidant potency of *Salvia miltiorrhiza* hydroalcoholic extract was determined using the FRAP method. The basis of this method relies on the extract’s ability to transform Fe3+ to Fe2+. tripyridyl-s-triazine (Merck, Darmstadt, Germany) was used as a reagent in acidic pH to react with Fe^2+^ and form Fe-TPTZ complex that was blue. A spectrophotometer (UV-2100 double beam scanning spectrophotometer, Lublin, Poland) at 593 nm was used to detect the Fe-TPTZ complex. Ferrous sulfate (FeSO4), at 25–1000 μmol / l was selected as standard. The result of the (FRAP) analysis was 24.73 × 10^2^ μmol FeSO4 equivalents/L.

### Animals and groups

The animal experiments were performed in compliance with the Ethical approval of the Animal Ethics Committee (code NO. LU. ACRA.2020.2, Lorestan University, Faculty of veterinary medicine) and the work has been reported in accordance with the ARRIVE guidelines (Animals in Research: Reporting In Vivo Experiments) [34]. Forty sexually mature non-pregnant female Wistar albino rats (250 ± 50 g), with the average age of 10 to 12 weeks, were employed in this work. The rats were obtained from Razi Herbal Medicines Research Center, Lorestan University of Medical Sciences, Khorramabad, Iran, and were housed in a well-ventilated rat house. Standard conditions for the maintenance of rats, including a 12-h light cycle, 65% ± 3% humidity and temperature of 22 ± 2 °C were prepared. Moreover, the rats were allowed to access tap water and Pellet chow freely. In compliance with institutional guidelines, all the animals received appropriate humane care. The rats (*n* = 40) were randomly divided into 5 groups of 8 per group:

- Group 1 (N): In the normal group (*n* = 8), no surgical procedure was done. This group was designed so that we could evaluate the baseline values of blood and antioxidant parameters.
- Group 2 (C): In the control group (*n* = 8), the adhesions were induced without any treatment.
- Group 3 (DW): The adhesions were induced, and they were treated with 3 ml of distilled water (*n* = 8).
- Group 4 (SME 1): The adhesions were induced, and they were treated with 3 ml of 1% *Salvia miltiorrhiza* extract (*n* = 8).
- Group 5 (SME 5): The adhesions were induced, and they were treated with 3 ml of 5% *Salvia miltiorrhiza* extract (*n* = 8).

### Surgical procedures

Anesthesia of rats was performed by intramuscular (IM) injection of ketamine | xylazine (100 mgkg^− 1^/ 10 mgkg^1^). The surgical site was shaved and the scrotum was disinfected using a 10% Povidone-iodine solution. An incision (3 cm length) was made in the linea alba to allow access to the abdominal area. Subsequently, to induce postoperative adhesions, 3 transverse and longitudinal incisions (length 1.5 cm) were made on the parietal peritoneum of the right abdominal wall, and using a scalpel blade No.11 a 1.5 × 1.5 piece was removed from the parietal peritoneum of the left abdominal wall. In the control group, only the adhesions were induced and no substance was injected into the peritoneum. In other groups, the previously mentioned substances were poured into the peritoneum before the surgical incision was closed. Eventually, the celiotomy incision was closed with 2–0 polydioxanone (PDS; Ethicon Inc., Somerville, NJ) applying a subcuticular suture pattern. Following the operation, the rats were kept in cages for 14 days under standard conditions. Buprenorphine (0.03 mgkg^− 1^q 12 h) was used as a postoperative analgesic for two consecutive days. After 14 days, the rats were euthanized by a specific dose of thiopental sodium (250 mgkg^− 1^, i.p. Panpharma, luitré, France), and celiotomy was performed in order to evaluate the adhesion bands in the experimental groups of the study. A sampling of adhesive bands was carried out and a 10% formalin buffer solution was utilized to fix tissue samples. No deaths or major complications were found during the experiments.

### Macroscopic evaluation (measurement of adhesions score)

**Fig. 1:**
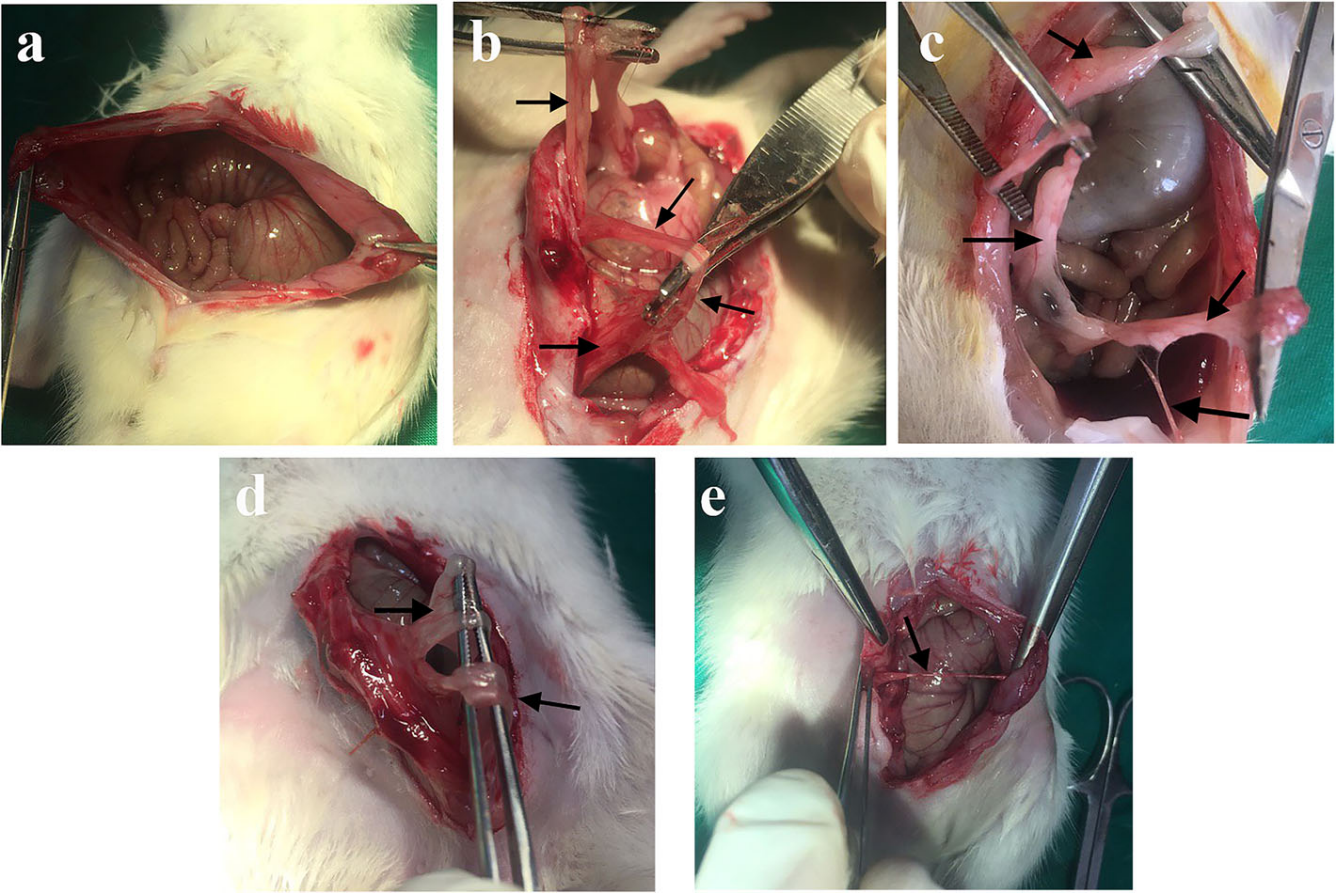
Macroscopic grading of adhesive bands in different groups of the study. a: Normal group without any adhesive band’s formation. b: control group with extensive and thick adhesive bands between viscera and/or abdominal wall. c: DW group with more than 2 bands between viscera or abdominal wall. d: The SME 1% treated group with two bands between the abdominal wall. e: The SME 5% with just one thin adhesive band between the abdominal wall. Black arrows indicate the adhesive bands

Fourteen days after the surgery, celiotomy was redone to assess the adhesion bands (Fig. 1). Macroscopic grading of the adhesion bands was performed using the method suggested by Nair et al. [35], as shown in Table 2.

**Table 2:** Adhesion scores based on the Nair et al. method

Score	Intensity of adhesions
0	No adhesion
1	One adhesion band, between viscera or from viscera to abdominal wall
2	Two adhesion bands, either between viscera or from viscera to abdominal wall
3	More than 2 bands, between viscera or from viscera to abdominal wall or whole intestines forming a mass without being adherent to abdominal wall
4	Extensive and thick adhesive bands and adhesions between viscera and/or abdominal wall

### Histology and immunohistochemistry

#### Histomorphological examinations

Tissue samples were primarily removed from the fixative solution (buffered formalin solution) and dehydration was performed at ascending levels of ethylene alcohol. Xylene was then used to clear tissue samples, and embedding in paraffin was done. 5 μm thick sections were provided with a rotary microtome (leica, rm2235, Nussloch, Germany). Afterwards, the sections were mounted on slides, and stained with hematoxylin and eosin (H&E).

The histomorphological changes were evaluated according to the intensity of inflammation and granulation tissue formation. The inflammation score was assessed as follows: 1 for mild inflammatory reaction, 2 for moderate inflammatory reaction, and 3 for severe inflammatory reaction. The fibrosis score was evaluated in 10 various microscopic fields. Granulation tissue formation was graded based on three degrees from 0 to 3 as follows: 0: no fibroblasts proliferation and angiogenesis | 1: from 1 to 10.99% | 2: from 11 to 50.99% and 3: from 51 to 100%.

#### Immunohistochemical evaluations

Peritoneal tissues with 3 μm thickness were deparaffinized and rehydrated. The samples were then immersed in a target retrieval solution (pH 9.0) and boiled water bath with temperature of 98 °C for 20 min to deliver unmasked antigens. In order to block endogenous peroxidase, the sections were treated with 3% H2O2 in PBS for 15 min. Subsequently, the sections were incubated with 5% normal rabbit sera in PBS for 20 min to block nonspecific background staining. The tissue sections were incubated in polyclonal rabbit anti CD31 (orb 10,314, Biorbyt, UK) for 1 h at 1:50 dilution, polyclonal goat anti-vimentin (orb 233,645, Biorbyt, UK) at 1:50 dilution, monoclonal rabbit anti-COX-2 (Biocare 306 A, USA) at 1:50, polyclonal rabbit anti-IL-1β (ab226918, Abcam, UK) at 1:100 dilution, and polyclonal rabbit anti-iNOS (NB300-605SS, Novus biologicals, USA) at 1:20 dilution. Thereafter, the sections were incubated in biotinylated goat anti-rabbit IgG (prediluted, Biocare, USA) for all markers, except for the vimentin that was incubated in rabbit anti-goat IgG (Sigma-Aldrich SAB 3700244) for 20 min, followed by the incubation with streptavidin horseradish peroxidase (sHRP) (prediluted, Biocare, USA) for 20 min. For the visualization of the antibody binding sites, a DAB solution was utilized. Ultimately, the sections were counterstained using the Mayer’s Hematoxylin (Bio optica, Italy). The score for IHC was graded on a scale of 0 to 3; 0, no reaction; 1, labeling ≤10% or mild as point reaction; 2, 10 up to 30% or moderate as a focal reaction, and 3, more than 30% or intensive as multifocal to diffuse reaction.

### Assessment of oxidative stress biomarkers

Malondialdehyde (MDA), glutathione peroxidase (GPx), catalase (CAT), and total antioxidant capacity (TAC) were evaluated in the peritoneal fluid with commercial biochemical kits (Asan, Khorramabad, Iran) according to the manufacture’s protocol.

### Analytical approach

In order to examine the SME effects on oxidative stress biomarkers, histopathological alterations and immunohistological markers, comparative analyses were conducted employing SPSS software (Version 25; IBM Corporation, Armonk, NY). Distribution of the data was checked based on the Kolmogorov-Smirnov single sample method. One-way ANOVA analysis and Tukey’s multiple comparison post hoc test were used to analyze the data with the normal distribution. The abnormal data were analyzed applying the Kruskal-Wallis test. The significance level was considered as *p* < 0.05.

## Results

### Macroscopic score

**Fig. 2:**
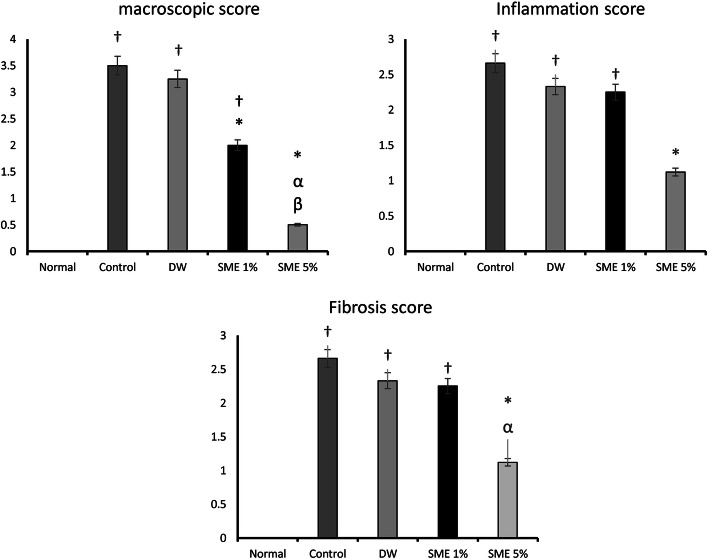
Macroscopic, inflammation, and granulation tissue formation score in different groups of the study. † indicates a significant difference with the normal group (*p* < 0.05). * indicates a significant difference with the control group (*p* < 0.05). α indicates a significant difference with the DW group (*p* < 0.05). β indicates a significant difference with the SME 1% group (*p* < 0.05)

Figure 2 provides the summary statistics for macroscopic scores of the peritoneal adhesions. As shown in the figure, a significant difference was observed between the normal group and the control group (*p* < 0.05). Additionally, the difference between the DW group and the normal group was significant (*p* < 0.05). No significant differences were found between the control group and the DW group (*p* > 0.05). The mean score for the SME 1% group was 2, which was significant compared to the control group (*p* < 0.05). SME 5% could remarkably decrease the score compared to the control group (*p* < 0.05). In the SME 5% group the macroscopic score was significantly lower than that in the SME 1% group (*p* < 0.05). Table 3 provides adhesion score of rats in each groups by detail. Adhesion bands were commonly found between two layers of the abdominal wall, however; in some of rats the bands were between abdominal wall and visceral organs such as stomach, spleen, intestines, and rarely bladder.

**Table 3:** Adhesion score of groups by detail

Rat Number	Groups				
	Normal	Control	DW	SME 1%	SME 5%
1	0	4	4	2	1
2	0	3	2	2	1
3	0	4	3	3	0
4	0	3	2	2	1
5	0	3	4	1	1
6	0	3	4	2	0
7	0	4	3	3	0
8	0	4	4	1	0

### Microscopic score

#### Inflammation score

Figure 2 presents the summary of statistics for the inflammation score in various experimental groups. As could be seen in Fig. 2, the control and DW groups reported significantly higher macroscopic scores than the normal group (*p* < 0.05). Even though SME 1% administration could not significantly decrease the inflammation score compared to the control group (*p* > 0.05), the SME 5% significantly diminished this score in comparison with the control group (*p* < 0.05). No significant difference was seen between the treatment groups concerning this score (*p* > 0.05).

#### Granulation tissue formation score

The results of the granulation tissue formation score (with or without fibrosis) are represented in Fig. 2. According to the graph, the differences between the control, distilled water, and SME 1% groups with the normal group were significant (*p* < 0.05). The SME 1% group reduced the granulation tissue score compared to the control group, yet the difference was not significant (*p* > 0.05). The treatment with SME 5% remarkably decreased granulation tissue score compared with the control group (*p* < 0.05). A comparison of different doses of *Salvia miltiorrhiza* hydroalcoholic extract revealed that granulation tissue score was lower in the SME 5% group although the difference was not significant (*p* > 0.05).

### Immunohistochemistry

**Fig. 3:**
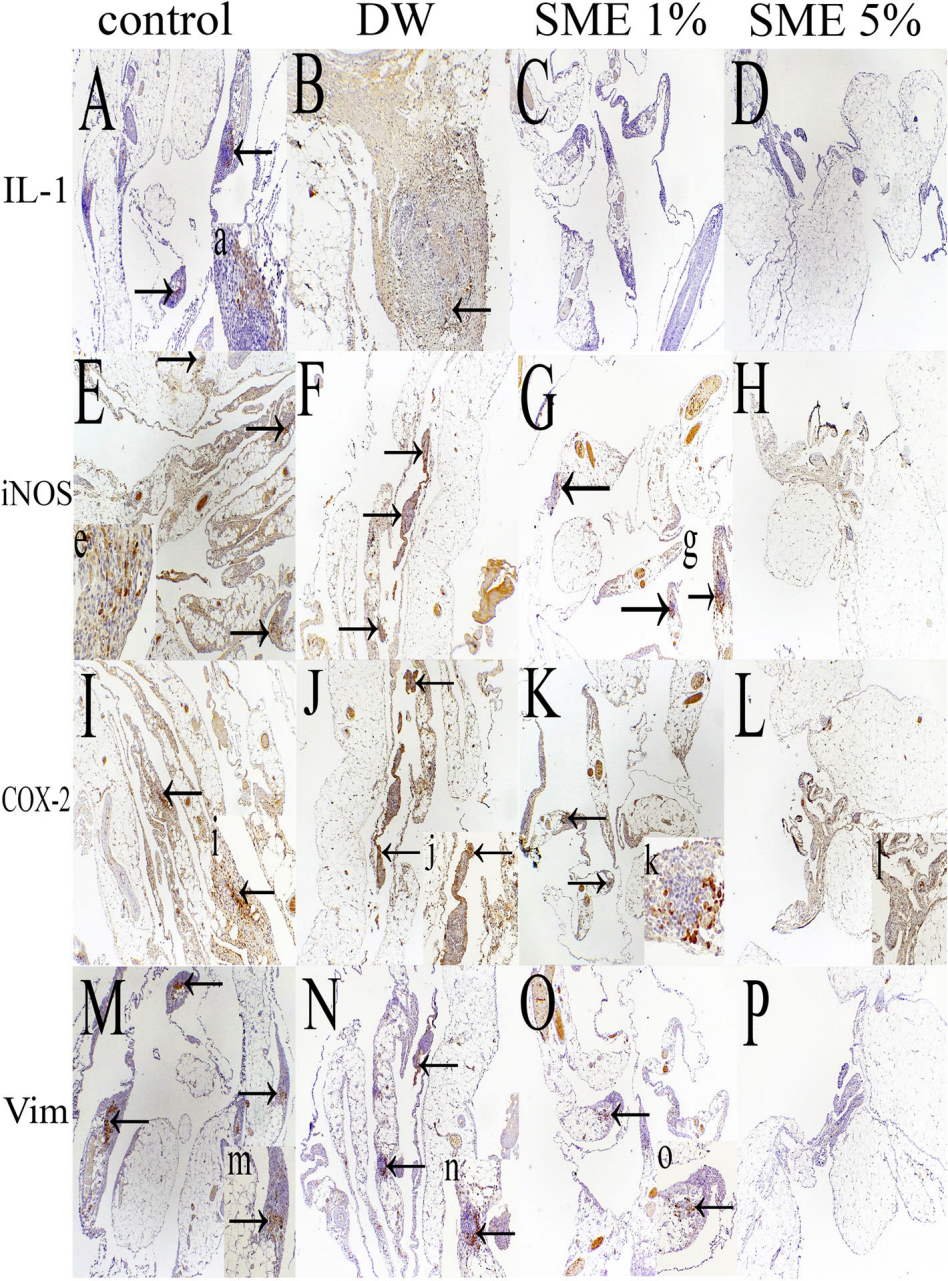
Immunohistochemistry of IL-1β protein (A-D). Multifocal inflammatory cells express inflammatory markers (arrows) in the control group (A). Inset a: higher magnification with intensive cytoplasmic expression. Immunostaining of mononuclear inflammatory cells (arrow) at the center of granuloma (B). No immunolabeling of inflammatory cells in SME1%(C) and SME 5% (D). The panel of anti iNOS antibody expression (E-H). Multifocal immunopositivity of inflammatory cells throughout the lesion (arrows) in the control group (E). Inset e: strong positive cells in higher magnification. Multifocal immunoexpression of marker (arrows) in peritoneal adhesion inflammatory cells (F). Multifocal with a lower population of cells’ immunolabeling (arrows) in contrast to the F group is presented (G). Inset g: aggregated intensive cells are immunostained (arrow). Highly dispersed mononuclear cells in a narrow rim of adhesive connective tissue (H). Immunostaining of COX-2 panel (I-L). The high density of inflammatory cells which have a cytoplasmic reaction (arrow) in control (I). Inset i: compressed and intense immunolabelled cells (arrow). Multifocal positive reaction in a condensed population of inflammatory cells (arrows) (J). Inset j: strong cytoplasmic staining (arrow). Moderate inflammatory cells but strongly expressed against antibody (arrows) (K). Inset k: cytoplasmic positive cells are demonstrated at the center of inflammation. Scattered point cells might be confined at the thin marginal surface (L). Inset l: undetectable inflammatory cells at higher magnification. The panel of anti-vimentin protein (M-P). Strong Multifocal reaction to antibody specified by arrows in the control group (M). Inset m: higher magnification of vimentin reaction in mesenchymal cells. Large numbers of connective tissue cells are immunolabeled against antibody (arrows) (N). Inset n: the cells are well demonstrated in this field (arrow). Very few populations of cells have a positive reaction to the antibody (arrow) (O). Inset o: More pronounced expressed cells are determined (arrow). No immunostaining cells are detected in a high dose of SME (P). A-P × 40. Inset: a, e, k, m (× 400) & g, i, j, l, n, o (× 100)

Except for CD31, other markers, including pro-inflammatory and vimentin, significantly reduced (vimentin [SME 1%], Cox-2, and iNOS) or were not even expressed (vimentin [SME 5%] and IL-1β) in the treatment groups. The results are depicted in Table 4 and Fig. 3.

**Table 4:** Comparison of the expression of immunohistochemical markers in different groups of the study

Group	Vimentin	IL1-β	COX-2	INOS
2 (C)	45.20 ± 5.80	23.00 ± 4.00	40.80 ± 5.80	39.80 ± 7.22
3 (DW)	24.00 ± 3.31^a^	25.00 ± 1.87	44.40 ± 4.56	44.60 ± 5.77
4 (SME 1%)	6.80 ± 1.30 ^a, b^	0.00 ± 0.00^a, b^	18.00 ± 5.87^a, b^	23.20 ± 4.43^a, b^
5 (SME 5%)	0.00 ± 0.00 ^a, b, c^	0.00 ± 0.00^a, b^	5.80 ± 1.92^a, b, c^	5.60 ± 1.34^a, b, c^

^a^*p* < 0.05 compared with the C group

^b^*p* < 0.05 compared with the DW group

^c^*p* < 0.05 compared with the SME 1% group

IHC sored based on

(No labeling = 0, labeling ≤10% = + 1, 10 up to 30% = + 2, and more than 30% = + 3)

### Oxidative stress biomarkers

**Fig. 4:**
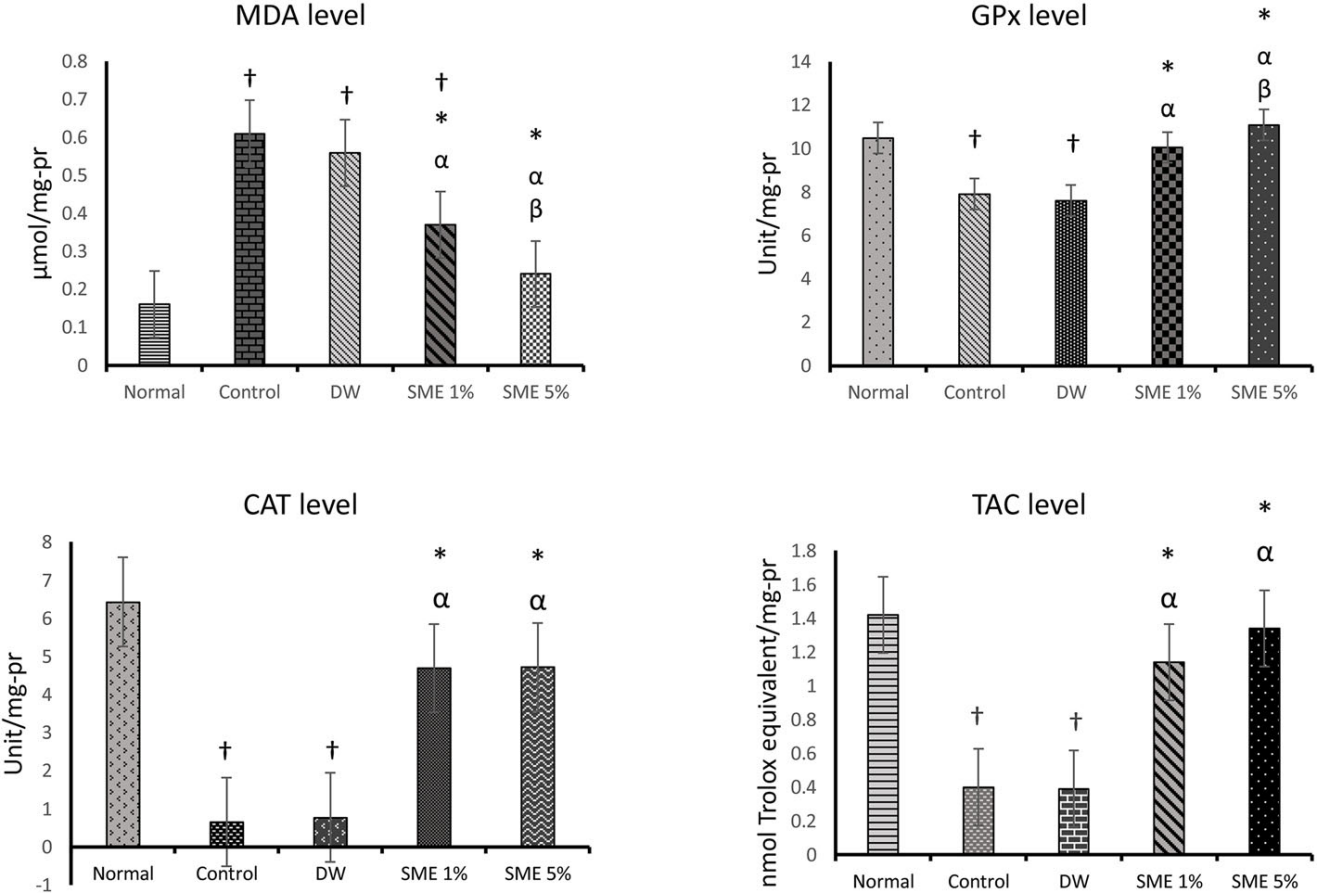
Oxidative stress biomarkers in experimental groups of the study. † indicates a significant difference with the normal group (*p* < 0.05). * indicates a significant difference with the control group (*p* < 0.05). α indicates a significant difference with the DW group (*p* < 0.05). β indicates a significant difference with the SME 1% group (*p* < 0.05)

Fig. 4 presents an overview of oxidative stress biomarkers in different groups of the study. The induction of peritoneal adhesions in the control group and the DW group remarkably increased the MDA level compared to the normal group (*p* < 0.05). The concentration of MDA in peritoneal fluid in both treatment groups significantly reduced compared to that in the control and distilled water groups (*p* < 0.05). A comparison of various doses of SME demonstrated that SME 5% administration significantly diminished MDA level compared with SME 1% group (*p* < 0.05).

GPx level in the control and DW groups was significantly lower than that in the normal group (*p* < 0.05). It was found that SME 1% and SME 5% significantly elevated the GPx level compared to the control group (*p* < 0.05).

Catalase activity significantly reduced in the control and DW groups compared to the normal group (*p* < 0.05). There was a substantial increase in the CAT level in the SME administered groups compared to that in the control group (*p* < 0.05). No significant difference was seen between low dosage and high dosage treatment groups (*p* > 0.05).

The induction of peritoneal adhesion decreased the TAC level in the control and DW groups compared to the normal group (*p* < 0.05). In the SME 1% and SME 5% groups the TAC levels were remarkably higher than that in the control group (*p* < 0.05). In all the oxidative stress biomarkers, no significant difference was observed between the control and DW group (*p* < 0.05).

## Discussion

Surgical procedures expose the abdominal area to the formation of peritoneal adhesions. The probable complications following the formation of adhesive bands include abdominal pain, small bowel obstruction, infertility, and difficulty in subsequent laparotomies [36]. The exact pathophysiology of adhesive band formation is not known, but studies suggest that healing of peritoneal damage causes these bands to form [37]. The peritoneal layer that covers the entire abdominal area has two layers, including a mesothelial layer and a sub mesothelial layer. Damage to the mesothelial layer of the peritoneum induces the inflammatory process, which is followed by fibrin formation, the most common pathogenesis of peritoneal adhesions [32]. One of the factors that stimulate the inflammatory process is oxygen-free radicals overproduction caused by hypoxia [38]. Hypoxia triggers oxidative stress, inflammation, and eventually, the formation of adhesive bands [39]. A lot of attempts have been made to help stop peritoneal adhesions after surgery and many studies have been done in this field, however, no way of prevention has been found yet to tackle these complications most effectively.

In view of the role of reactive oxygen species (ROS) in the formation of adhesions, in previous studies, several antioxidants have been used to inhibit the development of peritoneal intra-abdominal adhesions. Gallic Acid, Iranian propolis, green tea extract, *Rosmarinus officinalis* extract, resveratrol, honokiol, berberine hydrochloride, rhynchophylline, silymarin, breviscapine are antioxidants employed in previous studies to alleviate post-operation adhesions [40]. In the present study, hydroalcoholic extract of *Salvia miltiorrhiza*, which has strong antioxidant agents such as rosmarinic acid, was used to avoid the intraperitoneal adhesions development.

Previous studies have demonstrated that the required time for post-operation peritoneal adhesions formation is about 14 days, which begins from the first to the fifth day after a surgery [37]. In accordance with previous studies, in the present work, a 14-day period was considered for the formation of peritoneal adhesions in different experimental groups. Various methods are used to create the model of peritoneal adhesion in rats, for instance intestinal abrasion, large intestine anastomosis, ischemic buttons, and peritoneal incisions and removing a piece of the peritoneum [41]. In the current research, we employed the method of the peritoneal wall incision and removing a piece of peritoneum to induce adhesions, which is known to be one of the acceptable methods [42–44].

The macroscopic grading method used herein has been used extensively in previous researches [45]. Microscopic grading of fibrosis and inflammation was also employed for intraperitoneal adhesions in previous studies [43]. Concerning the groups treated with the SME with different concentrations, the macroscopic score and the severity of fibrosis and inflammation significantly reduced, which was notably lower in the SME groups than the control group. These findings are consistent with those of Parsaei et al., who reported that intraperitoneal administration of green tea extract decreased the macroscopic score of the adhesions and histological score of fibrosis and inflammation [43]. In another study, the use of gallic acid as a powerful antioxidant could significantly reduce the macroscopic score of adhesions [46].

It is well established that iNOS and COX-2 are produced in several inflammatory reactions, in which NO and PGs are produced by them respectively. Furthermore, previous studies have suggested a possible role for NO in COX-2 pathway regulation. Therefore, the anti-inflammatory effects of the iNOS inhibitors are due to blocking NO and PGs. However, the production of normal amounts of NO and PGs in different tissues in terms of blood supply is essential to maintain homeostasis against endogenous and exogenous damage. Thus, the inflammatory responses occur once NOS/COX cross-talk is disrupted, followed by the discharge of large amounts of NO and PGs, leading to inflammatory and degenerative processes [47].

The co-expression of iNOS and COX-2 in each group was consistent with the concept that iNOS and COX are engaged in a common track of pathophysiological conditions simultaneously. In addition, the activation of the iNOS gene, and consequently COX, is obtained through IL-1β, but not Il-1α. Accordingly, the axis of IL-1β -iNOS, and COX-2 is a growing consensus that stimulates inflammatory diseases [48]. Pata et al. examined the effect of inducible nitric oxide synthase on intra-abdominal adhesions and concluded that iNOS expression in the control group significantly increased compared to that in the treatment groups [49]. These results are in accordance with those observed in the present study.

We have not certainly recognized the direct mechanisms of action of SME to inhibit IL-1β release in order to prevent massive inflammation. However, the most probable mechanism is caspase-1 inhibition which consequently blocks the release of IL-1β. Certain drugs inhibit ATP-induced IL-1β release by NLRP3 inflammasome inactivation. A number of anti-inflammatory drugs act on cathepsin B activity which leads to the inhibition of the NLRP3 inflammasome and therefore, reduces IL-1β release. Understanding the exact mechanism with which SME prevents the release of interleukin-1β in this study requires further investigation [50].

Bi et al. (2017) evaluated the peripheral serotonin effects on the prevention of postoperative adhesions and concluded that in the serotonin treated group, the concentration of the MDA was remarkably less than that in the control group, and the levels of GSH and SOD were significantly higher than those in the control group [36]. Durmus et al. (2011) examined the biomarkers of oxidative stress, following vitamin E and selenium administration, in preventing postoperative adhesions. Their findings demonstrated that vitamin E injection significantly reduced the level of MDA, and vitamin E and selenium treatment rose the levels of CAT, GPx, GSH, but the difference was not significant [51]. Protective effects of resveratrol, which is a polyphenol produced by several plants, has been investigated in preventing peritoneal adhesions. Resveratrol administration significantly reduced MDA and increased GSH levels compared to the control group [52]. Askari et al. (2017) examined the effects of propolis on peritoneal adhesions and concluded that treatment with propolis significantly reduced MDA concentration in the peritoneal fluid and elevated GSH concentration compared to the control group [38]. In accordance with the previous studies, the present study illustrated that SME treatment groups significantly increased antioxidant enzymes and reduced the MDA level compared to the control group.

Overall, the results of this work implied that in the control group, more grade 4 adhesions were formed and the severity of granulation tissue formation and inflammation was high. The injection of distilled water did not have a positive effect on reducing postoperative adhesions. This finding is in agreement with Sortini et al.’s results (2006), which showed that lavage with distilled water after surgery did not diminish the adhesive bands [53]. In addition, Parsaei et al. (2013) concluded that distilled water had no positive effect in reducing peritoneal adhesions [43]. Between the two treatment groups, according to the measured parameters, the treatment group with 5% hydroalcoholic extract of *Salvia miltiorrhiza* revealed a greater effect on reducing postoperative adhesions.

## Conclusion

The present study aimed to determine the effect of *Salvia miltiorrhiza* hydroalcoholic extract on the prevention of postoperative adhesions in rats. Herein, we found that *Salvia miltiorrhiza* genrally alleviated peritoneal adhesion bands, oxidative stress biomarkers, and inflammatory cell reactions. A limitation of this study was that the gene expression of IL-1β, iNOS, COX-2 and vimentin were not studied.

## Data Availability

All data generated or analyzed during this study are included in this published article.

## Abbreviations

ROS: Reactive Oxygen Species
MDA: Malondialdehyde
GPx: Glutathione peroxidase
CAT: Catalase
TAC: Total antioxidant capacity
IHC: Immunohistochemistry
SME: *Salvia miltiorrhiza* extract
COX-2: Cyclooxygenase-2
iNOS: Inducible nitric oxide synthase
IL-1: Interleukin-1
IL-6: Interleukin-6
TNF-α: Tumor necrosis factor-alpha
MMPs: Matrix metalloproteinases
